# Distinct Functional Cortico-Striato-Thalamo-Cerebellar Networks in Genetic Generalized and Focal Epilepsies with Generalized Tonic-Clonic Seizures

**DOI:** 10.3390/jcm11061612

**Published:** 2022-03-15

**Authors:** Hsinyu Hsieh, Qiang Xu, Fang Yang, Qirui Zhang, Jingru Hao, Gaoping Liu, Ruoting Liu, Qianqian Yu, Zixuan Zhang, Wei Xing, Boris C. Bernhardt, Guangming Lu, Zhiqiang Zhang

**Affiliations:** 1 Department of Diagnostic Radiology, Jinling Hospital, Nanjing University School of Medicine, Nanjing 210093, China; fmrixiexy@126.com (H.H.); fmrixuq@126.com (Q.X.); yangfang021011@163.com (F.Y.); fmrizhangqr@126.com (Q.Z.); fmrihaojr@126.com (J.H.); fmriliugp@126.com (G.L.); fmriliurt@126.com (R.L.); fmriyuqq@126.com (Q.Y.); fmrizhangzx@126.com (Z.Z.); cjr.luguangming@vip.163.com (G.L.); 2 Department of Radiology, Third Affiliated Hospital of Soochow University/Changzhou First People’s Hospital, Changzhou 213004, China; suzhxingwei@126.com; 3 McConnell Brain Imaging Centre, Montreal Neurological Institute and Hospital, McGill University, 3801 University Street, Montreal, QC H3A 2B4, Canada; boris.bernhardt@gmail.com

**Keywords:** generalized tonic-clonic seizures, functional connectivity, support vector machine

## Abstract

This study aimed to delineate cortico-striato-thalamo-cerebellar network profiles based on static and dynamic connectivity analysis in genetic generalized and focal epilepsies with generalized tonic-clonic seizures, and to evaluate its potential for distinguishing these two epilepsy syndromes. A total of 342 individuals participated in the study (114 patients with genetic generalized epilepsy with generalized tonic-clonic seizures (GE-GTCS), and 114 age- and sex-matched patients with focal epilepsy with focal to bilateral tonic-clonic seizure (FE-FBTS), 114 healthy controls). Resting-state fMRI data were examined through static and dynamic functional connectivity (dFC) analyses, constructing cortico-striato-thalamo-cerebellar networks. Network patterns were compared between groups, and were correlated to epilepsy duration. A pattern-learning algorithm was applied to network features for classifying both epilepsy syndromes. FE-FBTS and GE-GTCS both presented with altered functional connectivity in subregions of the motor/premotor and somatosensory networks. Among these two groups, the connectivity within the cerebellum increased in the static, while the dFC variability decreased; conversely, the connectivity of the thalamus decreased in FE-FBTS and increased in GE-GTCS in the static state. Connectivity differences between patient groups were mainly located in the thalamus and cerebellum, and correlated with epilepsy duration. Support vector machine (SVM) classification had accuracies of 66.67%, 68.42%, and 77.19% when using static, dynamic, and combined approaches to categorize GE-GTCS and FE-GTCS. Network features with high discriminative ability predominated in the thalamic and cerebellar connectivities. The network embedding of the thalamus and cerebellum likely plays an important differential role in GE-GTCS and FE-FBTS, and could serve as an imaging biomarker for differential diagnosis.

## 1. Introduction

Generalized tonic-clonic seizure (GTCS), formerly known as ‘grand mal seizure’, is the most dangerous of the common seizure types. Typical symptoms include muscle stiffening, violent muscle rhythmical jerking of the entire body, and loss of consciousness [1]. GTCS can occur in genetic generalized epilepsy (GE-GTCS) or may arise from partial seizures with secondary generalization in focal epilepsy, referred to as focal to bilateral tonic-clonic seizure (FE-FBTS). In GE-GTCS, generalized symptoms occur at the beginning of seizure onset. Seizure activity involves the thalamus early on and rapidly engages bilaterally distributed brain networks [2]. In FE-FBTS, on the other hand, generalized symptoms always follow partial seizures. It is believed that focal seizure activity propagates to the thalamus, before activity spreads to the distributed bilateral brain via thalamo-cortical circuits [3,4]. In the clinic, these two epilepsy syndromes are often difficult to distinguish based on semiology only, which may lead to suboptimal pharmacotherapy calibration [5].

In epilepsy networks, structures in the basal ganglia and cerebellum, together with the thalamus and cortex, play important roles in propagation, termination, and modulation of seizure activity [6]. The striatum has been supposed to engage in modulation of epileptic discharge generalization [7] and regulation of tonic inhibition of the thalamus in GTCS [8]. The cerebellum also has modulatory effects on epileptic activity [9] and has been selected as a neurophysiological stimulation target for epilepsy therapy. All these nodes may contribute through seizure mechanisms via reciprocal and complex interactions. Thus, exploring the cortico-striato-thalamo-cerebellar network may broaden our understanding of neural mechanisms in GE-GTCS and FE-FBTS, and may have potential for distinguishing these epilepsy syndromes as imaging markers.

Multimodal brain imaging has been increasingly adopted for studying epilepsy networks. Using combined electroencephalography (EEG) and functional magnetic resonance imaging (fMRI) investigations, studies have demonstrated temporospatial patterns of blood oxygen level-dependent (BOLD) signal changes and functional connectivity in the striato-thalamo-cortical network, associated with different discharge states in idiopathic generalize epilepsy [10,11]. Our previous functional connectivity analyses have also delineated cortico-thalamic networks [12,13]. Structural covariance and diffusion MRI analyses have revealed structural substrates of the epilepsy network in idiopathic generalize epilepsy [9,14]. In parallel, studies using single photon emission computed tomography and fMRI have unraveled specific network patterns in FE-FBTS, suggesting specific pathophysiological processing underlying these two epilepsy syndromes [3,8]. However, the previous studies only addressed some specific nodes of the cortico-striato-thalamo-cerebellar network, and not the ensemble [12,13,14]. Moreover, they have not yet discriminated network patterns between these two epilepsy syndromes.

In this study, we adopted static and dynamic connectivity analyses on fMRI data, to map cortico-striato-thalamo-cerebellar networks in GE-GTCS and FE-FBTS using a winner-take-all strategy. Based on unraveling the specific alteration of network patterns in these two epilepsy syndromes, we further applied the network features for constructing a classification model. The study contributed imaging evidence for understanding the conception of epilepsy networks, and tested the feasibility of network markers for differential diagnosis between syndromes of generalized epilepsy.

## 2. Materials and Methods

### 2.1. Participants

A total of 228 patients (including 114 patients with GE-GTCS, and the same number of age- and sex-matched patients with FE-FBTS) were recruited from Jinling hospital between 2009 and 2018. All patients were diagnosed according to seizure symptoms, scalp-EEG, and therapeutic responses by two experienced neurologists with cross validation (F.Y. and G.C.), according to International League Against Epilepsy (ILAE) criteria [15]. For GE-GTCS, the patients had (1) presence of typical clinical symptoms of generalized tonic-clonic seizures, including tic of limbs, followed by a clonic phase of rhythmic jerking of the extremities, loss of consciousness during seizures without partial seizures and aura; (2) presence of generalized spike-and-wave or poly spike–wave discharge in their scalp EEG; (3) right-handedness. Sixty-three patients were taking anti-epileptic medications at the time of the scan, including: Sodium Valproate (35 cases), Carbamazepine (17 cases), Topiramate (13 cases), Phenytoin (10 cases), Phenobarbita (5 cases), Lamorgine (4 cases), Oxcarbazepine (3 cases), Clonazepam (3 cases), and Levetriaracetam (2 cases). For FE-FBTS, the patients presented (1) typical symptoms of focal frontal seizures, such as head and eye movement to one side, repetitive movements or abnormal body posturing accompanied by symptoms of secondary generalized tonic-clonic seizures, characterized by bilateral rigid tonic extremities followed by rhythmic clonic jerks, along with complete loss of consciousness; (2) focal frontal epileptic discharges on EEG; and (3) right-handedness. Ninety patients were taking anti-epileptic medications at the time of the scan: Sodium Valproate (50 cases), Topiramate (36 cases), Carbamazepine (33 cases), Phenytoin (14 cases), Phenobarbita (14 cases), Oxcarbazepine (13 cases), Lamorgine (5 cases), Levetriaracetam (4 cases), and Clonazepam (2 cases). Exclusion criteria of the two groups included (1) focal abnormality in routine structural MRI examinations; (2) history of epilepsy associated etiology; (3) mixed type of other genetic epilepsy, such as absence of seizures and juvenile myoclonic epilepsy; and (4) ages younger than 18 years or older than 55 years.

The same number of (*n* = 114) age- and sex-matched healthy subjects were recruited as controls. They had no history of psychiatric illnesses or other neurologic disorders. Considering that handedness may affect the symmetry of brain networks, all participants were right-handed. The study was approved by the Medical Ethics Committee in Affiliated Jinling Hospital, Medical School of Nanjing University. Written informed consent was obtained from each participant.

### 2.2. Data Acquisition

All data were collected on a 3 T Siemens Trio scanner with an eight-channel phased array head coil. Resting-state fMRI data were acquired using a gradient echo type echo planar imaging (GRE-EPI) sequence with the following parameters: TR/TE = 2000 ms/30 ms, FA = 90°, matrix = 64 × 64, FOV = 24 × 24 cm^2^, and slice thickness = 4 mm, slice gap = 0.4 mm, and 30 slices covering the whole brain. Each session contained 250 volumes. Each subject was instructed to relax, hold still, keep eyes closed, but stay awake during the resting-state functional MRI examination. High-spatial-resolution three-dimensional T1-weighted anatomic images were acquired in sagittal orientation, using a magnetization-prepared rapid gradient-echo sequence (TR/TE = 2300 ms/2.98 ms, flip angle= 9°, field of view = 256 × 256 mm^2^, matrix size = 256 × 256, slice thickness = 1 mm, 176 slices without intersection gap).

### 2.3. Data Preprocessing

MRI data preprocessing was carried out using pipeline of DPARSF V5.0 (http://rfmri.org/DPARSF (accessed on 15 January 2021)) based on toolkits from SPM12 (https://www.fil.ion.ucl.ac.uk/spm/software/spm12 (accessed on 27 January 2021)) [16]. The first 10 images were excluded, to ensure steady-state signal equilibrium. The remaining 240 scans were slice-time corrected and then realigned to the first volume, to correct for head motion. The data whose motion exceeded 1.5 mm or rotation exceeded 1.5° during scanning were excluded. All realigned images were spatially normalized to the Montreal Neurological Institute (MNI) template and resampled to the voxel size of 3 × 3 × 3 mm^3^. Then, the data were smoothed with an isotropic 4 mm FWHM Gaussian kernel. To remove spurious sources of variance, time series were preprocessed as follows: first, six head motion parameters, averaged signals from CSF and white matter, and global brain signal were regressed. Last, the data were bandpass filtered between 0.01 and 0.08 Hz, to remove the effects of very-low-frequency drift and high frequency noise using custom-made software.

### 2.4. Parcellation Maps of Cortico-Striatum, -Thalamus and-Cerebellum Connectivity

To investigate the specific patterns of functional relationships between the cortex and each subcortical structure, we first calculated maps of cortico-striatum, -thalamus, and-cerebellum connectivity. The cortex of each hemisphere was partitioned into five disjoint regions, in line with previous studies [12,17]; i.e., (1) prefrontal cortex, (2) motor/premotor cortex, (3) somatosensory cortex, (4) parietal/occipital cortex, and (5) temporal cortex. The masks of thalamus, striatum (including caudate, putamen and pallidum), and cerebellum were also acquired from an automated anatomic labeling template [18]. Static connectivity maps of the striatum, thalamus, and cerebellum were produced using partial correlation. In the calculation, the signals from other parcellations were set as covariates. Each voxel of the striatum, thalamus, and cerebellum was labeled according to the cortical lobe with the highest correlation coefficient, by applying a ‘winner-take-all’ (WTA) strategy [12,17]. Thus, the striatum, thalamus, and cerebellum could be separated into five sub-regions in each group.

**Figure 1:**
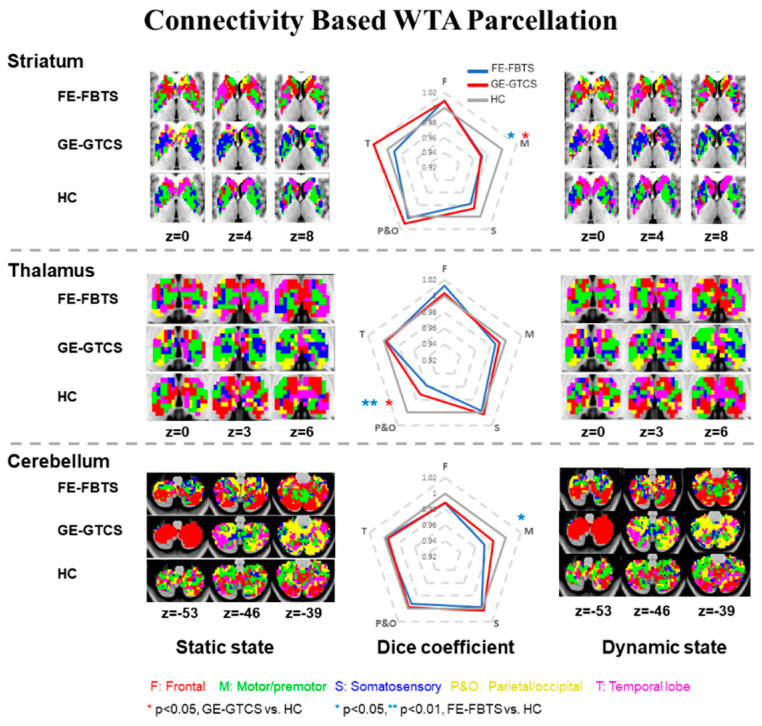
Maps of cortico-striato-thalamo-cerebellar connectivity network using the WTA strategy. Left column: maps of WTA in static state. Right column: maps of WTA in dynamic state. Middle column: the scale in each axis represents the dice coefficients between static and dynamic state to the corresponding parcellation, the value was standardized by dividing the dice coefficients in healthy controls. Red: voxels showing maximum value of the frontal lobe, Green: voxels showing maximum value of the motor/premotor lobe, Blue: voxels showing maximum value of the somatosensory cortex, Yellow: voxels showing maximum value of the parietal/occipital lobe, and violet: voxels showing maximum value of the temporal lobe.

Additionally, dynamic connectivity was also used for the parcellation of thalamus, striatum, and cerebellum, to observe the spatial similarity between the static and dynamic states. The dynamic connectivity was first calculated using a sliding-window method, with the sliding window size set as 50 TRs (100 s) and sliding step size as 5 TRs (10 s). The correlation coefficient of each cortical and subcortical structure in all windows was averaged. Connectivity was calculated between each voxel in different subcortical structure and each cortical parcellation, and was labeled according to the cortical lobe with the highest correlation coefficient, by applying the WTA strategy (Figure 1).

### 2.5. Construction of Cortico-Striato-Thalamo-Cerebellar Networks

To further investigate the interconnectivity strength between the cortical and subcortical structures, we constructed a cortico-striato-thalamo-cerebellar network in each group using static and dynamic connectivity analyses. A total of 20 regions-of-interest (ROIs) were selected, including the five cortical parcellations, and the corresponding regions of five cortical parcellations in the subcortical structures of striatum, thalamus, and cerebellum, through static WTA analysis of healthy controls. Signals were extracted and averaged from the 20 ROIs, for constructing a cortico-striato-thalamo-cerebellar network. Cortico-striato-thalamo-cerebellar networks were constructed using static and dynamic connectivity analysis, with a 20 × 20 matrix in each group.

Static functional connectivity was calculated via partial correlations across the entire time series. The correlation coefficient was converted to z-values using Fisher’s r-to-z transformation, to improve normality. Dynamic functional connectivity (dFC) was calculated using a sliding-window method [19]; here, the sliding window size was set as 50 TRs (100 s) and sliding step size as 5 TRs (10 s), in line with previous studies [20,21,22,23,24]. For the time series in each window, the dFC matrix was obtained. The variance of the correlation coefficient of the dFC matrix was estimated by calculating the standard deviation of z values at each ROI of the dFC matrix to assess dynamic functional connectivity flexibility.

### 2.6. Statistical Analysis

#### 2.6.1. Map Comparisons of Cortico-Striatum, Cortico-Thalamus, and Cortico-Cerebellar Connectivity

The WTA map showed the connectivity between the different subcortical nodes and the five cortical networks in each group. The spatial similarity of WTA maps between those with static (correlation coefficient) and dynamic (mean) connectivity was calculated through Dice coefficients and was shown in radar maps.

#### 2.6.2. Comparisons of Cortico-Striato-Thalamo-Cerebellar Networks

The cortico-striato-thalamo-cerebellar networks were represented as a 20 × 20 matrix, for both static and dynamic connectivity analysis. Groups were compared using two sample *t*-test and the findings were thresholded at *p* < 0.05, after FDR correction.

#### 2.6.3. Correlation with Clinical Variable of CORTICO-Striato-Thalamo-Cerebellar Networks

Pearson’s correlation analyses investigated the relationship between functional connectivity and disease duration in patients (*p* < 0.05, FDR correction). Linear interaction was calculated using SurfStat Toolbox [25] (https://mica-mni.github.io/surfstat/ (accessed on 18 May 2021)), to assess the relationship between functional connectivity and clinical variables of epilepsy duration among patient groups.

#### 2.6.4. Classification of Patients

Classification analysis was used to test the potential of cortico-striato-thalamo-cerebellar connectivity for discriminating between these two GTCSs. The features of the interregional connectivity in the cortico-striato-thalamo-cerebellar networks were sent as training data to classifiers. Three models, i.e., the static, dynamic connectivity, and combining static and dynamic connectivity, were constructed.

A linear support vector machine (SVM) classifier was adopted for classification. Since the number of features was much higher than the number of subjects used in classification (i.e., the features in static, dynamic connectivity, and combined static and dynamic connectivity were 400, 400, and 800, respectively), reducing the number of features not only sped up computation, but also improved the classification performance. In this study, the F score method was employed for feature ranking, which is simple, generally quite effective, and has been used in previous studies [26,27]. The larger the *F* score, the more discriminative the features.

A 10-fold cross-validation strategy was used to evaluate the performance of the classifiers. For each fold, we ranked the features according to their F scores, in descending order. It was worth noting that feature selection was only done for the training set of each fold, without using the information of the testing set, to avoid bias. Accuracy, sensitivity, and specificity were calculated to evaluate performance. To explore the dominant features associated with the cortico-striato-thalamo-cerebellar networks, the weight was multiplied by a label vector, indicating which group the scan belonged to. Each scan was then multiplied by the product of the weight and label and summed, resulting in a value for each functional connectivity, indicating its importance for group discrimination.

#### 2.6.5. Reproducibility Analysis

To test the reproducibility of our results, we carried out a split-half analysis. Specifically, we divided each participant group into two subgroups, matched for age and sex (HC1: 57 participants, twenty females, age: 24.76 ± 5.42 years; HC2: 57 participants, nineteen females, age: 25.38 ± 6.56 years; GE-GTCS1: 57 participants, twenty females, age: 26.29 ± 8.68 years; GE-GTCS2: 57 participants, nineteen females, age: 26.59 ± 8.8 years, FE-FBTS1: 57 participants, twenty females, age: 26.76 ± 7.65 years; FE-FBTS2: 57 participants, nineteen females, age: 25.78 ± 7.91 years) (all *p* > 0.6). For each subgroup, both static and dynamic connectivity networks were constructed and analyzed analogously to the aforementioned whole-group analysis. To determine whether there was a consistent topological organization, we computed Pearson’s correlations for the correlation patterns of both static and dynamic connectivity networks between HC1 and HC2 subgroups and between GE-GTCS1 and GE-GTCS2 subgroups and FE-FBTS1 and FE-FBTS2 subgroups.

Furthermore, three additional sliding window sizes (30 TRs (60 s), 60 TRs (120 s) and 100 TRs (200 s)) were utilized to validate the dFC findings.

## 3. Results

There was no difference in age and gender among GE-GTCS, FE-FBTS, and healthy controls, as well as no difference of disease duration and seizure frequency between GE-GTCS and FE-FBTS. All demographic and clinical data are summarized in Table 1.

### 3.1. Maps of Cortico-Striatum, -Thalamus, and-Cerebellum Connectivity

Each WTA map showed a pattern of connectivity to five cortical networks for each of the subcortical/cerebellar structures. Dice coefficient analysis revealed the high spatial similarity of WTA maps between static connectivity and dynamic connectivity. Computing the Dice coefficient between patients and controls, and GE-GTCS and FE-FBTS showed statically significant differences in motor cortex-striatum and parietal–occipital cortex-thalamus. For the cortical-cerebellum networks, FE-FBTS showed more connectivity in motor cortex-cerebellum (Figure 1; Table S1).

### 3.2. Group Comparisons of Cortico-Striato-Thalamo-Cerebellar Connectivity

Compared to controls, FE-FBTS and GE-GTCS both show altered functional connectivity, mainly correlated with the subregions of the motor/premotor and somatosensory networks. Among these two groups, the static connectivity of the cerebellum increased, while dFC variability decreased. In a static state, the connectivities between the striatum and thalamus and cerebellum were decreased in the FE-FBTS and GE-GTCS, while connectivity between cortex and thalamus were increased in GE-GTCS and decreased in FE-FBTS.

**Figure 2:**
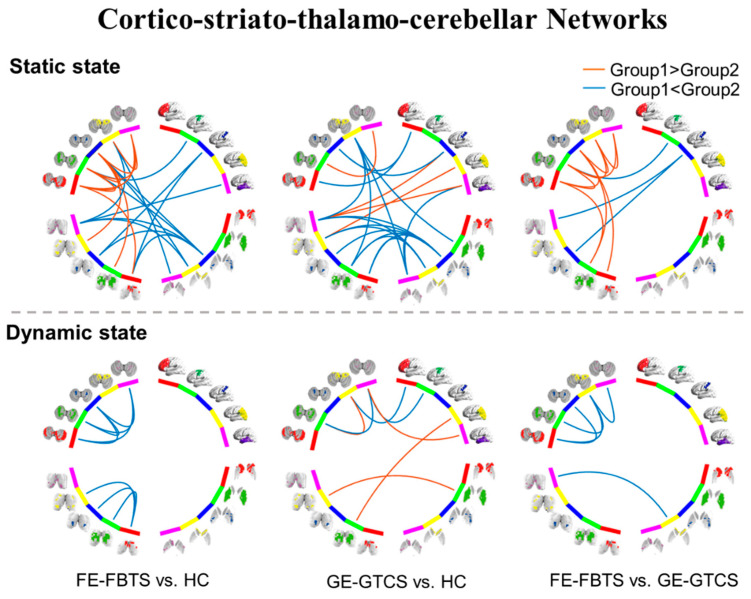
Group comparisons of cortico-striato-thalamo-cerebellar networks between three groups in static and dynamic state. Red: frontal lobe, Green: motor/premotor, Blue: somatosensory, Yellow: partial–occipital, Violet: temporal lobe.

Compared with GE-GTCS, the cerebellar connectivity was increased when analyzing connectivity statically in FE-FBTS, and decreased when analyzing connectivity dynamically. In addition, the connectivity between the cortex and the thalamus was decreased overall in FE-FBTS (Figure 2; Table S2).

### 3.3. Relation to Disease Duration

Epilepsy duration of FE-FBTS and GE-GTCS was negatively correlated with static, as well as dynamic, connectivity measures between the striatum and thalamus. Static functional connectivity between the somatosensory cortex and thalamus was negatively correlated with the epilepsy duration of FE-FBTS, while it was positively correlated with the epilepsy duration in GE-GTCS.

**Figure 3:**
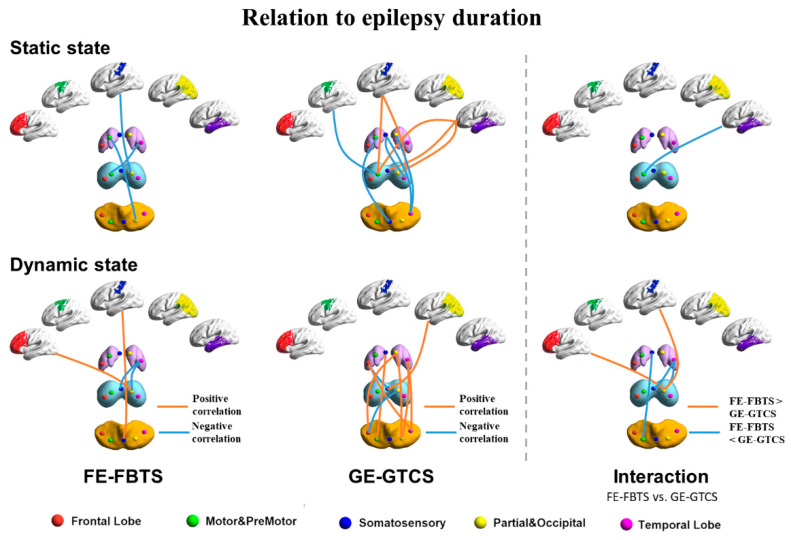
The correlation between functional connectivity networks and duration in the group of GE-GTCS, FE-FBTCS, and a comparison between the two patient groups in the static state and dynamic state. Red: frontal lobe, Green: motor/premotor, Blue: somatosensory, Yellow: partial–occipital, Violet: temporal lobe. Purple: striatum, Light blue: thalamus, Orange: cerebellum.

For comparison between patient groups, the disease had a more negative effect on the static functional connectivity between the cortex and thalamus, while having a positive effect on the dynamic functional connectivity in FE-FBTS, relative to GE-GTCS. Moreover, the disease had a more negative effect on the dynamic functional connectivity between the striatum, thalamus, and cerebellum in FE-FBTS relative to the GE-GTCS. The difference mainly correlated with the subregions of the somatosensory networks (Figure 3; Table S3).

### 3.4. Classification

**Figure 4:**
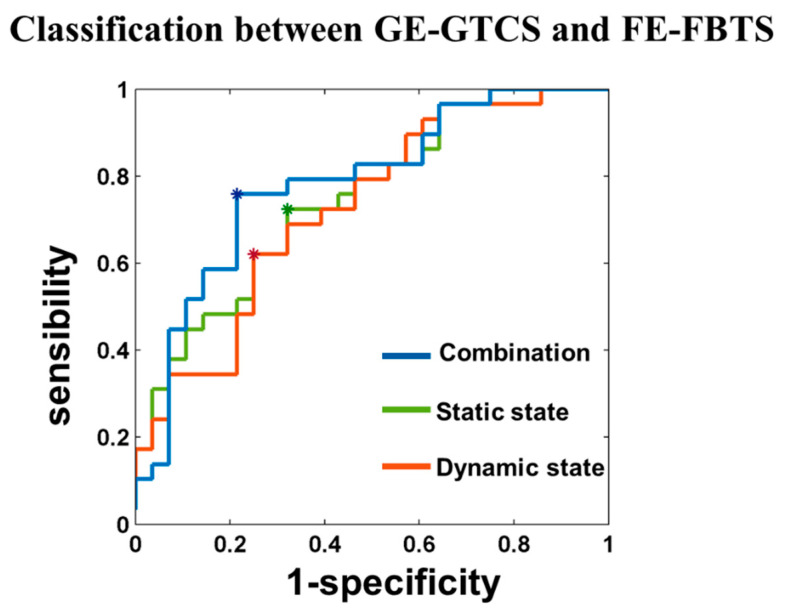
Classification between GE-GTCS and FE-FBTS. The accuracy, sensitivity, specificity, and AUC values between GE-GTCS and FE-GTCS were 66.67%, 72.74%, 67.86%, and 0.7475 when using static measures (Green); 68.42%, 62.07%, 75.00%, and 0.7278 when using dynamic measures (Orange); and 77.19%, 75.86%, 78.57%, and 0.7833 when combining static and dynamic functional connectivity metrics (Blue).

Classification showed the accuracy, sensitivity, specificity, and AUC values between GE-GTCS and FE-GTCS: 66.67%, 72.74%, 67.86%, and 0.7475 when using static measures; 68.42%, 62.07%, 75.00%, and 0.7278 when using dynamic measures; and 77.19%, 75.86%, 78.57%, and 0.7833 when combining static and dynamic functional connectivity metrics (Figure 4). The features with prominent discriminative abilities were mainly for connectivity associated with the thalamus and cerebellum (Table S4).

### 3.5. Reproducibility of Findings

We validated our main findings using different analysis strategies, involving split-half analysis and sliding window sizes. We found that with either a static or dynamic state, the previously constructed and matched split-half subgroups were all similar in topological organization for the cortico-striato-thalamo-cerebellar network (all *p* < 0.001) (Figure S1). Furthermore, the findings using different sliding window sizes were also maintained a high topological similarity (Figure S2).

## 4. Discussion

By constructing static and dynamic cortico-striato-thalamo-cerebellar networks using fMRI, we demonstrated distinct network patterns for the GE-GTCS and FE-FBTS. The changes in connectivity were mainly associated with the thalamus and cerebellum in both GE-GTCS and FE-FBTS, and were further correlated with the motor related network they belonged to. These connectivity changes were correlated with the disease duration. Importantly, the static and dynamic connectivity analyses complementarily depicted the features of the epilepsy network in different GTCS types, and contributed imaging markers for classifying GE-GTCS from FE-FBTS.

We first delineated the maps of specific connectivity with each cortical parcellation in the thalamus, striatum, and cerebellum using a WTA strategy. The study provided a comprehensive picture of connectivity alterations between cortical and subcortical structures in both epilepsy syndromes. Our results, thus, expanded previous imaging evidence showing atypical cerebellar and thalamo-cortical connectivity in GE-GTCS [9,12] and the striato-thalamic connectivity in FE-FBTS [8]. Network patterns were prominently changed in the thalamus and cerebellum in both GE-GTCS and FE-FBTS, primarily with cortical networks implicated in motor/premotor and somatosensory function. Abnormalities of the thalamus and cerebellum in patients with GE-GTCS and FE-FBTS had also been found in previous studies [3,28]. The thalamus has been thought to be critical for synchronizing abnormal cortical-subcortical electrical discharges, for generating tonic motor activity, and for producing impaired consciousness in epilepsy. In GE-GTCS, it engaged in generation of seizure activity [29,30] and was also the target for stimulating therapy [31]. In FE-FBTS, it engaged in propagation and synchronization of seizure activity [3]. Involvement of the thalamus supported its proposed role in seizure generalization. The cerebellum has been suggested to be involved in the regulation of epileptic activity [32,33]. Evidence from animal studies that demonstrated cerebellar involvement could lead to the termination of generalized spike and wave discharges, and the inhibition of epileptic activities more generally [34,35]. The cerebellar might undergo impairment from seizure propagation with a crossed cerebellar diaschisis mechanism [36], and presented with atrophy and atypical functioning in most epilepsy types [37,38,39]. Thus, our findings revealed that abnormal functional connectivity within the cerebellum of GE-GTCS and FE-FBTS might imply a potential modulatory effect of the cerebellum on the generation and propagation of epileptic activities. Motor abnormalities are a common clinical manifestation in GTCS, and could relate to our findings showing predominant involvement of motor/premotor and somatosensory cortical networks. Hyper-excitability of the motor cortex is known to be associated with clinical manifestations in patients with epilepsy [40]. Consistently, the present study demonstrated altered connectivities, mainly associated with the parcellations of motor/premotor and somatosensory network they belonged to, which was inferred to imply a disrupted inhibitory effect.

We found that disease duration was negatively correlated with the connectivity between striatum and thalamus in FE-FBTS and GE-GTCS. That is, the longer the patient had had the condition, the more functional connections between the striatum and the thalamus were reduced. These findings further suggest a weakening of the inhibition of striatum feedback [8]. Furthermore, the connectivity between the cortex and thalamus was negatively correlated with the disease duration of FE-FBTS, while being positively correlated in GE-GTCS, which might suggest that in those who had had the condition longer, the alternation of functional connectivity between the cortex and thalamus may further serve to affect synchrony. Thus, the differences in disease effects between patients mainly occurred in the thalamus, suggesting that the thalamus plays different roles in these different types of GTCS.

Moreover, our results suggested that the combination of static and dFC features improved classification between different types of GTCSs. Recent studies have highlighted the abundant information contained within the dynamic characteristics of functional connectivity [41,42]. Dynamic functional connectivity has been applied to characterize the inherent dynamic properties of brain networks in epilepsy patients and provided information about how the connectivity changed over time, rather than representing the mean functional connectivity [43]. Dynamic connectivity measures functional connectivity in each time window, and likely captures important information that is missed in a static FC approach [44]. The present findings showed that combining static and dFC achieved a significant improvement in the accuracy rate of classification compared to either single feature, suggesting that dFC can add information for classification. Regarding the distribution of the features, network features from the thalamus and cerebellum were prominent discriminators. Previous studies have shown changed imaging measures in the thalamus and cerebellum, with reduced cerebral flow in GE-GTCS and increased in FE-FBTS [3,28], suggesting the key roles of these structures in the process of these GTCS syndromes. The present findings may be regarded as evidence for understanding the mechanisms of seizure generation, and may also help to identify targets for prevention or treatment.

Several limitations in this work should be noted. First, follow-up data for longitudinal analysis are currently lacking, which might be helpful for validating the modulating results of disease duration. Second, we only used epilepsy duration for describing the progression of epilepsy and did not take into account the frequency or total number of seizure incidences [45]. Third, the possible effect of partial seizures in FE-FBTS were not taken as a factor for analysis. Moreover, the current work lacked other independent validation data to test the generality of classification. Furthermore, we only focused on the striatum, thalamus, and cerebellum, and other structures that may be important in the circuitry were not taken into account.

## 5. Conclusions

By constructing static and dynamic cortico-striato-thalamo-cerebellar networks using fMRI, we demonstrated distinct network patterns between GE-GTCS and FE-FBTS. The study contributed imaging evidence for understanding the conception of epilepsy networks, and tested the feasibility of using network markers for differentiating GE-GTCS from FE-FBTS. The network embedding of the thalamus and cerebellum likely plays an important differential role in GE-GTCS and FE-FBTS, and could serve as an imaging biomarker for differential diagnosis.

## Figures and Tables

**Table 1:** Demographic and clinical information.

	Age (Mean ± SD) Years	Gender Male/Female	Durations (Mean ± SD) Years	Seizure Frequency (per Month)
GE-GTCS	26.44 ± 8.74	75/39	7.98 ± 8.32	2.212 ± 10.76
FE-FBTS	26.27 ± 7.78	75/39	9.42 ± 6.56	2.34 ± 6.21
HC	25.07 ± 5.99	75/39	-	-
Statistical Value	F = 0.957	Chi-square < 0.001	t = 0.187	t = 0.105
*p* Value	0.385 ^a^	1 ^b^	0.574 ^c^	0.916 ^c^

Abbreviations: ^a^: one-way ANOVA, ^b^: chi-square, ^c^: two-sample *t*-test. SD: standard deviation, GE-GTCS: genetic epilepsy with generalized tonic-clonic seizures, FE-FBTS: focal to bilateral tonic-clonic seizure, HC: healthy controls.

## Data Availability

The data that support the findings of this study are available from the corresponding author, Zhiqiang Zhang, on reasonable request.

## Supplementary Materials

The following supporting information can be downloaded at https://www.mdpi.com/article/10.3390/jcm11061612/s1, Figure S1: Evaluation of the reproducibility of the results for the cortico-striato-thalamo-cerebellar connectivity network (all *p* < 0.001); Figure S2: Robustness analysis for the dynamic connectivity network (all *p* < 0.001); Table S1: Pairwise comparison results of dice coefficient of Cortex ROIs in striatum, thalamus, cerebellum among three groups; Table S2: Pairwise comparison results of Cortico- Striato-Thalamo-Cerebellar networks in static and dynamic state among three groups; Table S3: Correlation between functional connectivity networks and duration in static and dynamic state; Table S4: The top 5 discriminative features of the classification between GE-GTCS and FE-FBTS of static, dynamic and combined state.
