# Supplementary material for: Distinct Functional Cortico-Striato-Thalamo-Cerebellar Networks in Genetic Generalized and Focal Epilepsies with Generalized Tonic-Clonic Seizures
Source: J Clin Med. 2022 Mar 15;11(6):1612. doi: 10.3390/jcm11061612 (PMC8951449; doi:10.3390/jcm11061612)
Supplement: Supplementary file 1 [file jcm-11-01612-s001.zip › jcm-1589684-supplementary.pdf]

Supplemental Material

Table S1. Pairwise comparison results of dice coefficient of Cortex ROIs in striatum, thalamus, cerebellum among three groups.

	**GE-GTCS vs. HC**			**FE-FBTS vs. HC**		
**Seed ROIs**	**GE-GTCS**	**HC**	**p value**	FE-FBTS	HC	p value
Striatum						
Motor cortex	0.8147	0.8396	**0.03**	0.8160	0.8396	**0.03**
Thalamus						
Parietal&Occipital lobe	0.7637	0.7858	**0.04**	0.7528	0.7858	**0.007**
Cerebellum						
Motor cortex	-	-	-	0.777	0.7998	**0.02**

Abbreviations: GE-GTCS, genetic epilepsy with generalized tonic-clonic seizures; FE-FBTS, focal to bilateral tonic-clonic seizure; HC, healthy Controls.

Table S2. Pairwise comparison results of Cortico- Striato-Thalamo-Cerebellar networks in static and dynamic state among three groups.

		**FE-FBTS vs. HC**	**GE-GTCS vs. HC**	**FE-FBTS vs. GE-GTCS**	**FE-FBTS vs. HC**	**GE-GTCS vs. HC**	**FE-FBTS vs. GE-GTCS**
		**Static state (t value/p value)**			**Dynamic state (t value/p value)**		
Within Thalamus							
Frontal lobe(Thalamus)	Motor&Premoto (Thalamus)	1.369/0.172	0.318/0.751	1.034/0.302	**-2.065/0.04**	-1.006/0.316	-1.071/0.286
Frontal lobe(Thalamus)	Somatosensory(Thalamus)	0.438/0.662	0.827/0.409	-0.385/0.701	**-2.444/0.015**	-1.679/0.095	-0.82/0.413
Frontal lobe(Thalamus)	Parietal&Occipital(Thalamus)	0.668/0.505	1.022/0.308	-0.359/0.72	**-2.353/0.02**	-1.485/0.139	-0.899/0.37
Within Cerebellum							
Frontal lobe (Cerebellum)	Motor&Premoto(Cerebellum)	**3.094/0.002**	1.044/0.298	**2.225/0.027**	-1.082/0.281	0.547/0.585	-1.651/0.100
Frontal lobe (Cerebellum)	Somatosensory (Cerebellum)	**2.608/0.010**	0.284/0.777	**2.369/0.019**	**-2.073/0.04**	0.498/0.619	**-2.595/0.010**
Frontal lobe (Cerebellum)	Parietal&Occipital (Cerebellum)	1.154/0.250	-0.512/0.609	1.819/0.071	-0.999/0.319	1.336/0.183	**-2.264/0.025**
Frontal lobe (Cerebellum)	Temporal lobe(Cerebellum)	**3.153/0.002**	1.324/0.187	1.89/0.06	**-2.053/0.041**	-0.568/0.57	-1.43/0.154
Motor&Premoto (Cerebellum)	Somatosensory (Cerebellum)	**2.453/0.015**	0.818/0.415	1.565/0.119	**-2.879/0.004**	-1.613/0.108	-1.198/0.232
Motor&Premoto (Cerebellum)	Parietal&Occipital (Cerebellum)	1.732/0.085	-1.449/0.149	**3.508/0.001**	-0.461/0.645	**2.318/0.022**	**-2.701/0.008**
Motor&Premoto (Cerebellum)	Temporal lobe(Cerebellum)	**4.645/<0.001**	**2.658/0.009**	**2.093/0.038**	**-3.781/<0.001**	**-2.461/0.015**	-1.642/0.102
Somatosensory (Cerebellum)	Parietal&Occipital (Cerebellum)	0.740/0.460	-1.760/0.080	**2.478/0.014**	-0.453/0.651	1.512/0.132	-1.955/0.052
Somatosensory (Cerebellum)	Temporal lobe(Cerebellum)	**2.711/0.007**	1.424/0.156	1.129/0.26	**-3.058/0.003**	-1.342/0.181	-1.554/0.122
Parietal&Occipital(Cerebellum)	Temporal lobe(Cerebellum)	**2.704/0.007**	0.033/0.973	**2.672/0.008**	-1.892/0.06	0.936/0.351	**-2.716/0.007**
Between Cortical and Subcortical regions							
Frontal lobe(Cortex)	Frontal lobe(Cerebellum)	-1.918/0.057	**-2.505/0.013**	0.632/0.528	1.673/0.096	1.656/0.099	0.112/0.911
Frontal lobe(Cortex)	Somatosensory (Cerebellum)	-1.972/0.050	**-2.210/0.028**	0.284/0.777	-0.475/0.635	1.27/0.205	-1.719/0.087
Frontal lobe(Cortex)	Parietal&Occipital (Cerebellum)	-0.568/0.571	-1.521/0.130	0.885/0.377	-1.866/0.064	**-2.328/0.021**	0.425/0.671
Motor&Premoto(Cortex)	Motor&Premoto (Cerebellum)	**-3.600/<0.001/**	-0.709/0.479	**-2.761/0.006**	0.292/0.771	-0.631/0.529	0.946/0.345
Motor&Premoto (Cortex)	Temporal lobe (Cerebellum)	-0.129/0.897	1.653/0.100	-1.87/0.063	-1.095/0.275	**-2.159/0.032**	1.161/0.247
Somatosensory(Cortex)	Frontal lobe(Thalamus)	**-2.348/0.020**	-0.490/0.625	-1.771/0.078	-1.657/0.099	-0.201/0.841	-1.322/0.188
Somatosensory(Cortex)	Somatosensory (Thalamus)	**-2.789/0.006**	0.587/0.558	**-3.144/0.002**	0.297/0.767	0.851/0.396	-0.56/0.576
Somatosensory(Cortex)	Temporal lobe(Thalamus)	0.038/0.970	**2.371/0.019**	**-2.339/0.02**	0.42/0.675	-0.023/0.982	0.426/0.671
Somatosensory (Cortex)	Somatosensory(Cerebellum)	-1.903/0.059	**-2.289/0.023**	0.296/0.767	0.349/0.727	-1.025/0.307	1.39/0.166
Parietal&Occipital(Cortex)	Motor&Premoto(Thalamus)	-1.208/0.229	-0.807/0.420	-0.421/0.674	1.078/0.283	**2.631/0.009**	-1.489/0.138
Parietal&Occipital(Cortex)	Parietal&Occipital(Thalamus)	-1.380/0.169	**2.120/0.035**	-0.572/0.568	-0.755/0.451	1.262/0.209	-1.773/0.078
Temporal lobe(Cortex)	Temporal lobe (Thalamus)	**-2.374/0.019**	**2.386/0.018**	-0.23/0.818	0.275/0.784	0.098/0.922	0.18/0.858
Between Striatum and Thalamus							
Motor&Premoto (Striatum)	Parietal&Occipital (Thalamus)	2.148/0.330	1.343/0.181	0.893/0.373	1.157/0.249	**2.034/0.043**	-0.857/0.392
Somatosensory (Striatum)	Frontal lobe (Thalamus)	**-2.711/0.007**	**-3.278/0.001**	0.325/0.745	-0.656/0.512	-0.133/0.894	-0.531/0.596
Somatosensory (Striatum)	Motor&Premoto (Thalamus)	-1.946/0.053	**-2.573/0.011**	0.337/0.736	0.744/0.458	1.149/0.252	-0.355/0.723
Somatosensory (Striatum)	Somatosensory (Thalamus)	**-2.325/0.021**	**-2.271/0.024**	-0.117/0.907	-0.993/0.322	-0.583/0.56	-0.369/0.713
Somatosensory (Striatum)	Parietal&Occipital (Thalamus)	-1.435/0.153	**-2.260/0.025**	0.734/0.464	-0.402/0.688	-0.923/0.357	0.541/0.589

Somatosensory (Striatum)	Temporal lobe (Thalamus)	**-2.049/0.042**	**-2.061/0.041**	-0.26/0.795	-1.203/0.23	-0.831/0.407	-0.354/0.724
Parietal&Occipital (Striatum)	Frontal lobe (Thalamus)	-1.7/0.091	**-2.169/0.031**	0.453/0.651	-0.527/0.599	-0.184/0.855	-0.316/0.752
Parietal&Occipital (Striatum)	Motor&Premoto (Thalamus)	-1.704/0.09	**-2.191/0.030**	0.34/0.734	0.675/0.501	1.416/0.159	-0.64/0.523
Parietal&Occipital (Striatum)	Parietal&Occipital (Thalamus)	-1.412/0.16	**-2.108/0.36**	0.585/0.559	-0.048/0.961	0.219/0.827	-0.262/0.794
Parietal&Occipital (Striatum)	Temporal lobe (Thalamus)	**-2.115/0.036**	**-3.175/0.002**	0.8/0.425	-0.445/0.657	1.607/0.11	**-1.992/0.048**
Between Thalamus and Cerebellum							
Frontal lobe(Thalamus)	Frontal lobe (Cerebellum)	**2.01/0.046**	0.054/0.957	1.909/0.058	0.647/0.518	-0.48/0.632	1.145/0.254
Frontal lobe(Thalamus)	Somatosensory (Cerebellum)	1.696/0.091	-0.895/0.372	**2.54/0.012**	-0.524/0.601	-0.704/0.482	0.165/0.869
Motor&Premoto(Thalamus)	Frontal lobe (Cerebellum)	1.158/0.248	-1.033/0.303	**2.179/0.031**	-0.356/0.722	-0.789/0.431	0.368/0.713
Motor&Premoto(Thalamus)	Somatosensory (Cerebellum)	**2.06/0.041-**	-0.516/0.607	**2.456/0.015**	-0.627/0.532	0.031/0.976	-0.66/0.51
Motor&Premoto (Thalamus)	Parietal&Occipital(Cerebellum)	-1.179/0.24-	**-3.548/<0.001**	1.915/0.057	1.231/0.22	0.643/0.521	0.659/0.511
Parietal&Occipital(Thalamus)	Somatosensory (Cerebellum)	**2.399/0.017**	1.702/0.090	0.798/0.426	0.177/0.859	-1.152/0.251	1.251/0.213
Temporal lobe (Thalamus)	Parietal&Occipital(Cerebellum)	**-2.451/0.015**	**-2.792/0.006**	0.043/0.966	1.765/0.079	0.956/0.34	0.973/0.332
Between Striatum and Cerebellum							
Motor&Premoto(Striatum)	Parietal&Occipital(Cerebellum)	0.201/0.841	**-2.320/0.021**	0.107/0.915	-0.136/0.892	0.855/0.394	-1.046/0.297
Somatosensory (Striatum)	Motor&Premoto (Cerebellum)	**-2.08/0.039**	-1.364/0.174	-0.865/0.388	0.693/0.489	1.093/0.276	-0.457/0.648
Somatosensory (Striatum)	Somatosensory (Cerebellum)	**-1.989/0.048**	-0.991/0.323	-1.097/0.274	-0.781/0.436	-1.558/0.121	0.693/0.489
Parietal&Occipital(Striatum)	Somatosensory (Cerebellum)	**-2.052/0.042**	-1.396/0.164	-0.733/0.464	0.183/0.855	-0.921/0.358	1.083/0.28
Temporal lobe (Striatum)	Parietal&Occipital(Cerebellum)	**-2.099/0.037**	**-2.801/0.006**	0.562/0.575	1.289/0.199	1.045/0.297	0.265/0.791

Note: In the first and second columns of the table, the index used the form of Seed ROI (Target Region), for example, Frontal lobe (Thalamus) represented the subregion of thalamus whose seed region was frontal lobe. The Bold represents a statistically significant difference ($p < 0.05$).

Table S3. Correlation between functional connectivity networks and duration in static and dynamic state.

		**FE-FBTS**	**GE-GTCS**	**FE-FBTS *vs.* GE-GTCS**	**FE-FBTS**	**GE-GTCS**	**FE-FBTS *vs.* GE-GTCS**
			**Static state**			**Dynamic state**	
		r/p	r/p	t/p	r/p	r/p	t/p
Cortic- Subcortex connectivity							
Frontal lobe (Cortex)	Parietal&Occipital (Thalamus)	-0.195/0.059	-0.095/0.364	1.487/0.069	**0.264/0.010**	0.085/0.417	**1.675/0.048**
Motor&Premoto (Cortex)	Motor&Premoto (Thalamus)	-0.052/0.619	**-0.216/0.037**	0.855/0.189	0.082/0.429	-0.049/0.642	0.916/0.180
Somatosensory (Cortex)	Motor&Premoto (Thalamus)	0.171/0.098	**0.206/0.047**	0.018/0.493	0.059/0.570	0.010/0.923	0.415/0.339
Somatosensory (Cortex)	Parietal&Occipital (Thalamus)	**-0.214/0.038**	0.065/0.537	-1.282/0.101	**0.268/0.009**	-0.097/0.353	**2.716/0.004**
Somatosensory (Cortex)	Temporal lobe (Thalamus)	0.180/0.081	**0.226/0.030**	-0.017/0.493	-0.043/0.679	0.084/0.422	-0.825/0.205
Parietal&Occipital (Cortex)	Somatosensory (Thalamus)	-0.036//0.728	-0.204/0.050	0.931/0.176	0.101/0.330	**0.235/0.023**	-2.206/0.104
Temporal lobe (Cortex)	Motor&Premoto (Thalamus)	0.049/0.636	**0.308/0.003**	**-1.659/0.049**	**-0.253/0.013**	0.042/0.691	-2.12/0.108
Temporal lobe (Cortex)	Somatosensory (Thalamus)	0.134/0.197	**0.229/0.027**	-0.507/0.306	-0.135/0.191	0.052/0.691	-1.377/0.085
Striato- Thalamus connectivity							
Frontal lobe (Striatum)	Somatosensory (Thalamus)	0.130/0.209	-0.071/0.499	1.397/0.082	0.000/0.997	**-0.265/0.010**	1.58/0.058
Somatosensory (Striatum)	Temporal lobe (Thalamus)	-0.095/0.358	**-0.280/0.007**	0.532/0.298	0.066/0.526	0.096/0.362	-0.094/0.463
Temporal lobe (Striatum)	Motor&Premoto (Thalamus)	**-0.216/0.035**	0.038/0.714	-1.420/0.079	0.162/0.118	0.015/0.886	1.184/0.119
Temporal lobe (Striatum)	Somatosensory (Thalamus)	0.122/0.238	0.007/0.947	0.865/0.194	**-0.210/0.042**	-0.080/0.447	**-1.971/0.025**
Temporal lobe (Striatum)	Parietal&Occipital (Thalamus)	0.101/0.330	-0.048/0.650	1.040/0.150	**-0.334/0.001**	0.043/0.681	**-2.370/0.009**
Striato- Cerebellum connectivity							
Frontal lobe (Striatum)	Temporal lobe (Cerebellum)	0.079/0.449	-0.129/0.217	1.346/0.090	0.043/0.680	**0.221/0.033**	-0.924/0.178
Motor&Premoto (Striatum)	Somatosensory (Cerebellum)	0.044/0.673	**-0.24/0.021**	1.729/0.403	0.071/0.495	0.071/0.497	0.178/0.429
Motor&Premoto (Striatum)	Parietal&Occipital(Cerebellum)	**-0.23/0.025**	0.102/0.328	-2.493/0.070	0.024/0.815	0.092/0.380	-0.308/0.379
Somatosensory (Striatum)	Motor&Premoto (Cerebellum)	-0.042/0.684	-0.068/0.518	0.006/0.498	-0.190/0.065	**0.275/0.008**	**-3.159/<0.001**
Somatosensory (Striatum)	Temporal lobe (Cerebellum)	-0.009/0.935	**-0.248/0.017**	1.243/0.108	-0.019/0.855	0.094/0.370	-0.692/0.245
Parietal&Occipital (Striatum)	Frontal lobe (Cerebellum)	0.057/0.581	-0.092/0.381	0.979/0.164	0.052/0.616	**0.281/0.006**	-1.243/0.108
Parietal&Occipital (Striatum)	Parietal&Occipital(Cerebellum)	0.000/1.000	-0.132/0.206	0.797/0.213	0.159/0.123	**0.285/0.006**	-0.869/0.193
Parietal&Occipital (Striatum)	Temporal lobe (Cerebellum)	0.040/0.699	**-0.269/0.009**	1.767/0.309	0.049/0.636	0.020/0.849	0.263/0.396
Temporal lobe (Striatum)	Parietal&Occipital (Cerebellum)	-0.071/0.494	0.091/0.387	-1.076/0.142	0.097/0.347	**0.268/0.009**	-0.785/0.217
Temporal lobe (Striatum)	Temporal lobe (Cerebellum)	0.114/0.270	-0.038/0.720	1.126/0.131	0.018/0.866	**0.315/0.002**	1.738/0.402
Between Thalamus and Cerebellum connectivity							
Parietal&Occipital (Thalamus)	Frontal lobe (Cerebellum)	-0.115/0.268	-0.150/0.152	-0.048/0.481	-0.079/0.477	**-0.255/0.014**	0.866/0.194

Note: In the first and second columns of the table, the index used the form of Seed ROI (Target Region), for example, Parietal&Occipital (Thalamus) represented the subregion of thalamus whose seed region was parietal and occipital lobe. The Bold represents a statistically significant difference ($p < 0.05$).

Table S4. The top 5 discriminative features of the classification between GE-GTCS and FE-FBTS of static, dynamic and combined state.

Ranking	Static state	Dynamic state	Combined state
1	Motor&Premoto(Cerebellum)-Parietal&Occipital(Cerebellum)	Parietal&Occipital(Cerebellum)-Temporal lobe(Cerebellum)	Motor&Premoto(Cerebellum) (static state)-Somatosensory(Cerebellum) (static state)
2	Somatosensory(Cortex)-Somatosensory(Thalamus)	Motor&Premoto(Cortex)-Parietal&Occipital(Striatum)	Somatosensory(Cortex) (dynamic state)-Somatosensory(Thalamus) (dynamic state)
3	Motor&Premoto(Cortex)-Motor&Premoto(Cerebellum)	Temporal lobe(Thalamus)-Parietal&Occipital(Cerebellum)	Somatosensory(Cerebellum) (dynamic state)-Parietal&Occipital (Cerebellum) (dynamic state)
4	Frontal lobe(Thalamus)-Somatosensory(Cerebellum)	Motor&Premoto(Cerebellum)-Parietal&Occipital(Cerebellum)	Parietal&Occipital (Cerebellum) (dynamic state)-Temporal lobe (Cerebellum) (dynamic state)
5	Motor&Premoto(Thalamus)-Somatosensory(Cerebellum)	Parietal&Occipital(Cortex)-Parietal&Occipital(Thalamus)	Frontal lobe(Thalamus) (dynamic state)-Somatosensory(Cerebellum) (dynamic state)

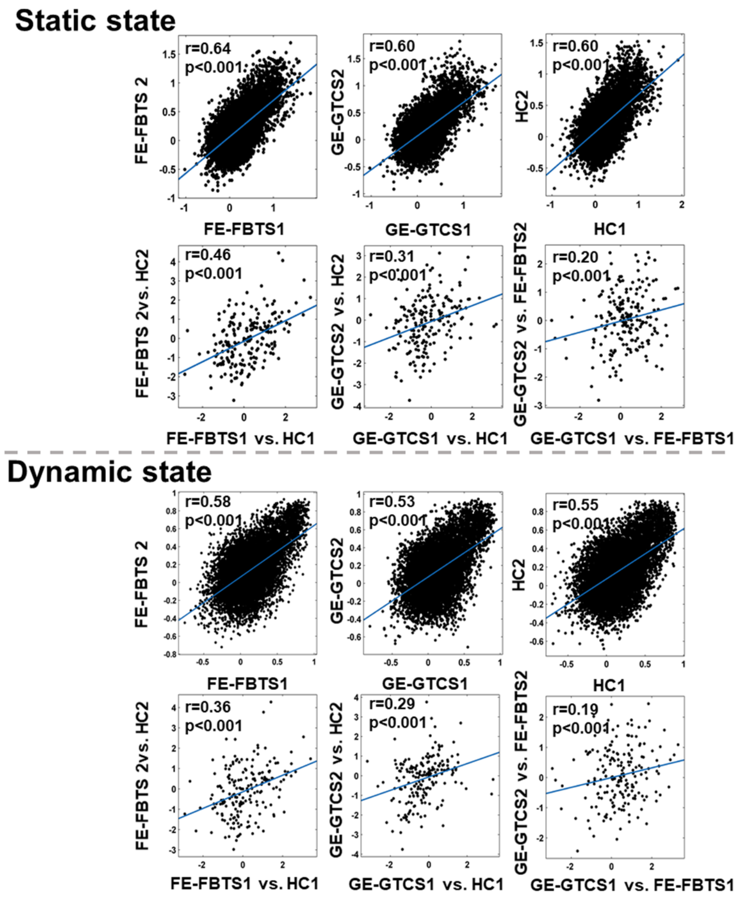

Figure S1. Evaluation of the reproducibility of the results for the cortico-striato-thalamo-cerebellar connectivity network (all $p < 0.001$).

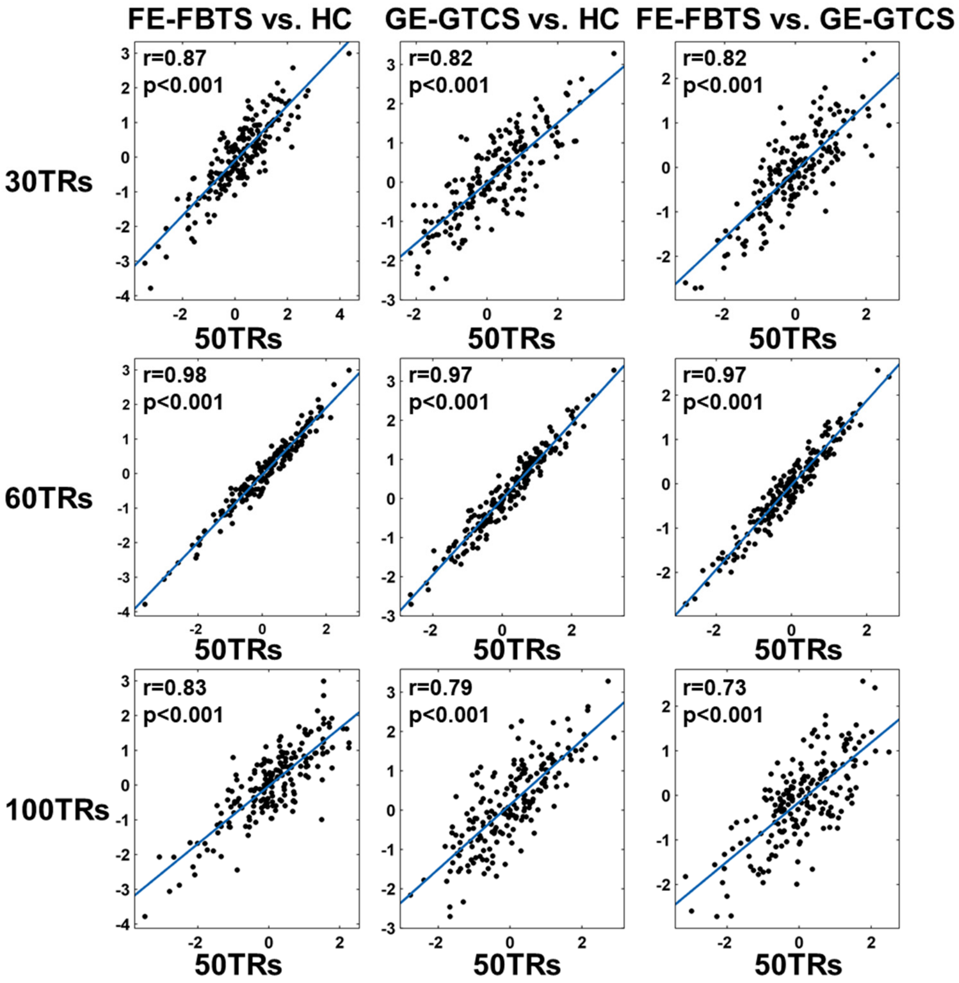

Figure S2. Robustness analysis for the dynamic connectivity network (all $p < 0.001$).
